# Hiding in Plain Sight: An Atypical Presentation of the Uncommon Merkel Cell Carcinoma

**DOI:** 10.7759/cureus.55613

**Published:** 2024-03-06

**Authors:** Elisha Myers, Matthew Uhde

**Affiliations:** 1 Dermatology, Florida Atlantic University Charles E. Schmidt College of Medicine, Boca Raton, USA; 2 Dermatology, Palm Beach Dermatology Group, Delray Beach, USA

**Keywords:** cutaneous apudoma, primary small cell carcinoma, trabecular carcinoma of the skin, skin nodule, cutaneous neoplasm, merkel cell carcinoma

## Abstract

Merkel cell carcinoma (MCC) is a cutaneous neoplasm that is challenging to diagnose secondary to its rarity. We report a case involving a 76-year-old Caucasian female with a seemingly benign skin nodule on her right forearm. Histopathological analysis revealed characteristics of MCC, including uniform round cells with minimal cytoplasm and fine granular chromatin. Immunohistochemical staining confirmed insulinoma-associated protein 1 (INSM1) positivity, a marker with high sensitivity and specificity in localized MCC diagnosis. The subsequent treatment plan involved wide local excision, sentinel lymph node evaluation, and radiation therapy, aligning with therapeutic standards for MCC. Negative positron emission tomography (PET) scans and follow-up for one year have demonstrated no evidence of recurrence or additional lesions. This case demonstrates the challenges in diagnosing MCC and the need for histopathological and immunohistochemical assessments for an accurate diagnosis. Diagnostic markers, INSM1, are important distinguishing factors between MCC and other skin cancers. In conclusion, our case contributes to the literature in diagnosing MCC and successful treatment, while emphasizing the need for immunohistochemical markers for accurate diagnosis and guiding therapeutic decisions.

## Introduction

Merkel cell carcinoma (MCC), also known as primary neuroendocrine carcinoma of the skin, trabecular carcinoma of the skin, primary small cell carcinoma of the skin, or cutaneous apudoma, is a rare cutaneous neoplasm. MCC was first described by Cyril Toker, a pathologist, in 1972, after he observed an aggressive skin cancer displaying dense-core granules, a distinctive feature only known to be found in Merkel cells [1]. Later in 1985, several neuroendocrine similarities were also uncovered between the tumor and Merkel cells [2].

The exact etiology of MCC remains unknown, but contrary to its name, MCC is not believed to originate from Merkel cells [3,4]. Merkel cells are specialized cells found in the skin’s basal layer and are specialized receptor cells of touch [5]. MCC and Merkel cells share morphologic, immunohistochemical, and ultrasound features; however, there is little evidence for a direct histogenetic relationship between them [6]. In 2008, an association between MCC and polyomavirus was reported [7]. In addition to the polyomavirus infection that is typically seen in immunocompromised patients, several other risk factors, including exposure to UV radiation, immunocompromised states, and fair skin types, have been identified in the development of MCC [8,9].

MCC predominantly affects older adults, with a median age of diagnosis at 73 in males and 76 in females [8,9]. As the condition is relatively rare, diagnosis is often clinically challenging and often associated with delayed recognition. Consequently, by the time the diagnosis is established, the tumor has typically already metastasized [3]. However, the incidence of MCC continues to rise, with the number of cases expected to be more than 3000 annually in the United States by 2025 [10]. This increase in prevalence is possibly secondary to increased rates of detection and an aging population.

Clinically, MCC typically manifests as a painless, firm, rapid growth with red to purple hues [5]. It is usually distinct and does not resemble other benign lesions. Diagnosis typically requires a clinical biopsy [5]. Dermoscopic examination of MCC often shows deep cherry-red coloring, linear irregular vessels, milky pink areas, architectural disorder, and a lack of pigmented structures [11], aiding in early recognition and management in an aging population where MCC incidence is increasing. MCC is characterized by uniform monotonous, round cells exhibiting minimal cytoplasm and fine granular chromatin [3]. Immunohistochemical staining for MCC commonly involves several markers, including cytokeratin 20 (CK20), neuron-specific enolase (NSE), and insulinoma-associated protein 1 (INSM1) [12]. INSM1 has emerged as a valuable marker for neuroendocrine differentiation and is increasingly used in the diagnosis of MCC due to its high specificity and sensitivity for MCC [12,13].

Our case report describes a presentation of MCC in a 76-year-old Caucasian female, initially presenting with a seemingly benign skin-colored nodule on her right forearm. Pathological analysis confirmed MCC based on histological features and positive staining for INSM1.

## Case presentation

**Figure 1:**
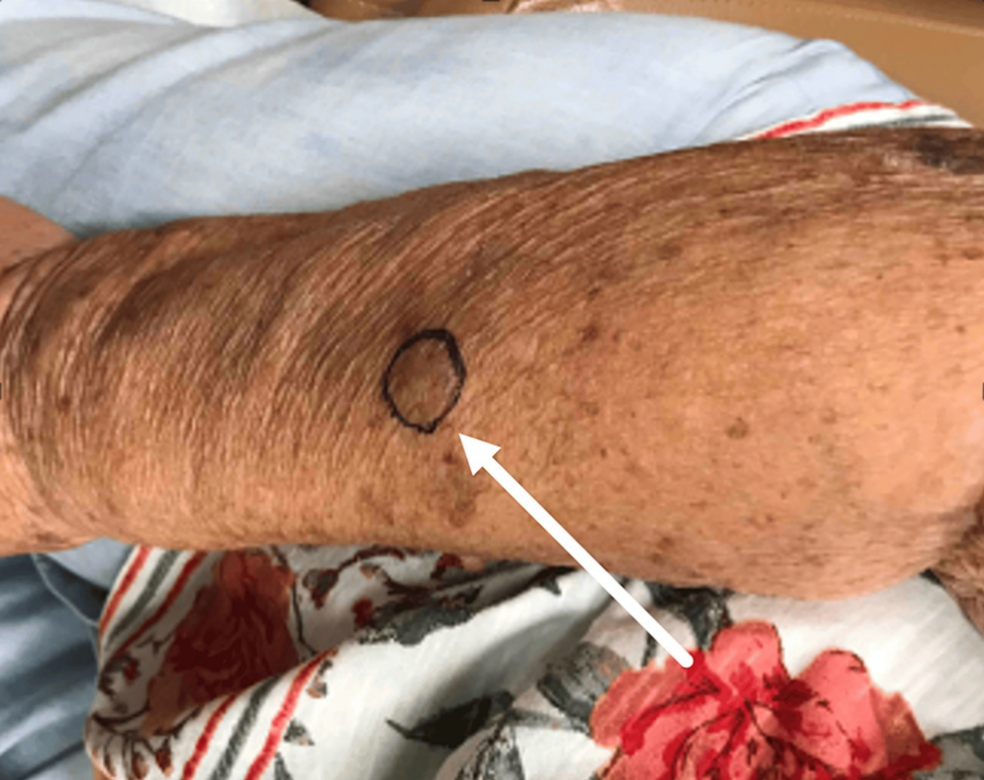
Skin-colored nodule on the right forearm. Biopsied lesions are photographed prior to biopsy.

A 76-year-old Caucasian female presented to a dermatology clinic with complaints of a two-centimeter skin-colored nodule on her right forearm, which had been present for over a month (Figure 1). The lesion was soft, mobile, and non-tender on the exam. At that time, the patient was also noted to have a 1.2-centimeter skin-colored, non-tender, soft, mobile mass on her left forearm. The patient’s past medical history was unremarkable, and no other complaints were reported. Both masses were excised to rule out any concerning conditions and sent to pathology. The differential diagnosis included a lipoma or a possible adnexal tumor, such as an epidermal cyst.

**Figure 2:**
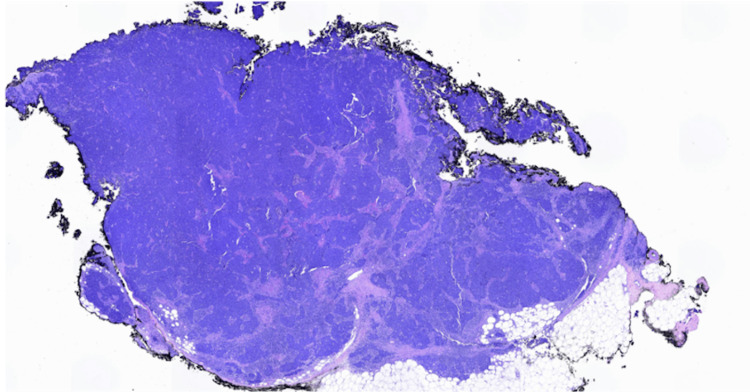
Histopathology of right forearm lesion. Hematoxylin and eosin stain, 10x objective.

**Figure 3:**
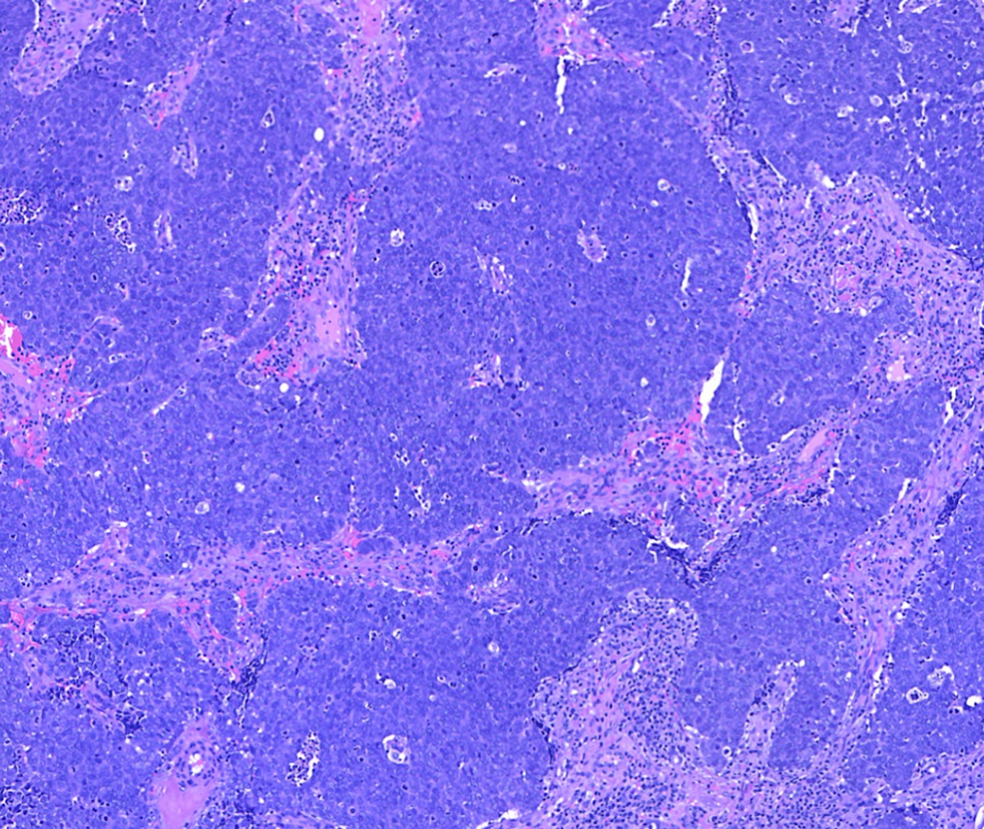
Histopathology of right forearm lesion. Hematoxylin and eosin stain, 40x objective.

**Figure 4:**
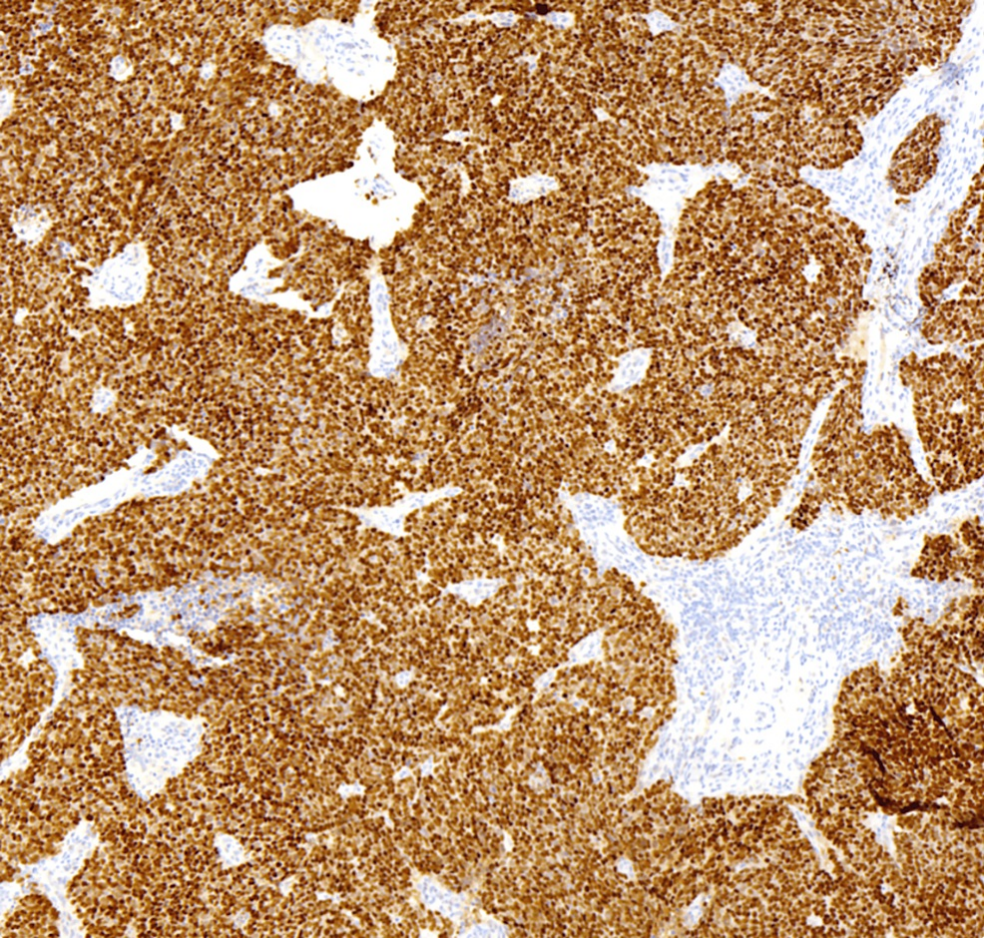
Immunohistochemical analysis positive staining for insulinoma-associated protein 1 (INSM1). Hematoxylin and eosin stain, 40x objective.

**Figure 5:**
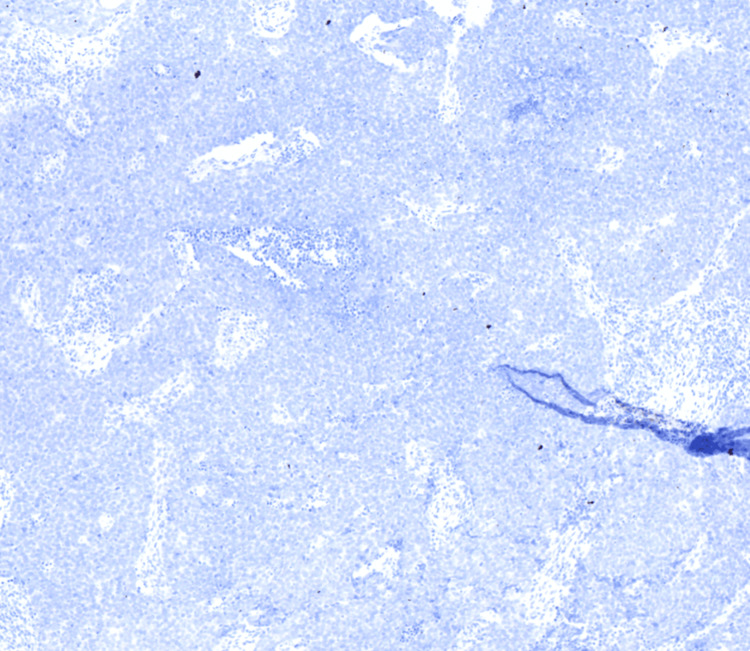
Immunohistochemical analysis negative staining for thyroid transcription factor-1. Hematoxylin and eosin stain, 40x objective.

Pathology results of the left arm biopsy confirmed the presence of a lipoma, whereas findings from the biopsy of the lesion on the right forearm were consistent with MCC. Histopathological examination of the right forearm biopsy unveiled an abundance of blue, consistently uniform, monotonous, round cells characterized by scant cytoplasm and fine granular chromatin (Figures 2, 3). Immunohistochemical analysis demonstrated positive staining for INSM1 (Figure 4) and negative staining for thyroid transcription factor-1 (TTF-1) (Figure 5), indicating a neuroendocrine carcinoma consistent with MCC.

Following the biopsy results, the patient underwent a comprehensive skin examination at the clinic, revealing no other noteworthy lesions. Subsequently, the patient was referred to a local surgeon and oncologist, where she underwent a two-centimeter local excision with sentinel lymph node evaluation. They radiated the surgical site and the nodal basin, delivering a total dose of 50 Gy over 25 treatments. Pathology revealed negative histologic margins, sentinel lymph nodes were negative, and positron emission tomography (PET) scans showed no signs of metastasis. Over the past year, the patient has undergone regular skin examinations, demonstrating no evidence of local recurrence, additional lesions, or lymphadenopathy.

## Discussion

MCC poses a diagnostic challenge due to its rarity [5]. This case, involving a 76-year-old Caucasian female with a seemingly benign skin-colored nodule, demonstrates the clinical challenges in MCC diagnosis. The lesion’s soft, mobile, and non-tender presentation highlights the deceptive nature of MCC, as it usually resembles a more obvious aggressive skin condition. The initial differential diagnosis of the skin-colored nodule included a lipoma or adnexal tumor, demonstrating the similarities to frequently seen benign dermatological conditions. The diagnosis was only able to be differentiated from the benign conditions with histopathological analysis, demonstrating the importance of histopathological and immunohistochemical assessments in establishing an accurate diagnosis.

Histopathological analysis of the biopsy revealed characteristic features of MCC, with blue, uniform round cells with minimal cytoplasm and fine granular chromatin. Immunohistochemical staining was positive for INSM1, which has emerged in the literature as a highly specific and sensitive marker for identifying localized MCC [14]. INSM-1 is a transcription factor and a marker for neuroendocrine differentiation. When MCC is localized, INSM-1 is particularly useful for distinguishing MCC from other skin cancers. Importantly, in patients with metastatic disease, INSM-1 cannot be used to distinguish MCC from other neuroendocrine tumors. The expression of INSM1 in MCC may also have prognostic implications, with further research on this still ongoing [15].

The patient’s subsequent wide local excision with negative sentinel lymph node evaluation, followed by a radiation therapy treatment plan, was aimed at achieving both local control and minimizing potential systemic spread. This treatment plan aligns with therapeutic standards for MCC [16]. The negative PET scans in combination with the year-long follow-up without evidence of recurrence or additional lesions are positive indicators for treatment success. Clinical trials continue to explore new treatment options, including therapeutic vaccines, cellular immunotherapies, and new histone deacetylase (HDAC) inhibitors such as domatinostat [16].

The increasing prevalence of MCC emphasizes the importance of immunohistochemical markers like INSM1 in achieving accurate diagnoses. With ongoing research, further insights into the prognostic implications of these markers may contribute to refining therapeutic approaches and improving patient outcomes in the management of MCC.

## Conclusions

In summary, our case report highlights the diagnostic challenges associated with Merkel cell carcinoma (MCC), a rare cutaneous neoplasm. The case of a 76-year-old Caucasian female with a seemingly benign skin nodule highlights the importance of histopathological and immunohistochemical assessments in achieving an accurate diagnosis. The positive staining for INSM1, a specific marker for localized MCC, guided the subsequent treatment plan involving wide local excision, sentinel lymph node evaluation, and radiation therapy. With ongoing research and insights into immunohistochemical markers, particularly INSM1, there is potential for refining therapeutic approaches and improving outcomes in the management of MCC amid its increasing prevalence.
